# Functional characterization and evolution of PTH/PTHrP receptors: insights from the chicken

**DOI:** 10.1186/1471-2148-12-110

**Published:** 2012-07-06

**Authors:** Pedro LC Pinheiro, João CR Cardoso, Deborah M Power, Adelino V M Canário

**Affiliations:** 1 Centre of Marine Sciences, Comparative Molecular Endocrinology, Universidade do Algarve, Campus de Gambelas, 8005-139, Faro, Portugal

## Abstract

**Background:**

The parathyroid hormone (PTH)-family consists of a group of structurally related factors that regulate calcium and bone homeostasis and are also involved in development of organs such as the heart, mammary gland and immune system. They interact with specific members of family 2 B1 G-protein coupled receptors (GPCRs), which have been characterised in teleosts and mammals. Two PTH/PTHrP receptors, PTH1R and PTH2R exist in mammals and in teleost fish a further receptor PTH3R has also been identified. Recently in chicken, PTH-family members involved in calcium transport were characterized and specific PTHRs are suggested to exist although they have not yet been isolated or functionally characterized. The aim of this study is to further explore the evolution and function of the vertebrate PTH/PTHrP system through the isolation, phylogenetic analysis and functional characterization of the chicken receptors.

**Results:**

Two PTHRs were isolated in chicken and sequence comparison and phylogenetic analysis indicate that the chicken receptors correspond to PTH1R and PTH3R, which emerged prior to the teleost/tetrapod divergence since they are present in cartilaginous fish. The vertebrate PTH2R receptor and its ligand TIP39 have been lost from bird genomes. Chicken PTH1R and PTH3R have a divergent and widespread tissue expression and are also evident in very early embryonic stages of development. Receptor stimulation studies using HEK293 cells stably expressing the chicken PTH1R and PTH3R and monitoring cAMP production revealed they are activated by chicken 1–34 N-terminal PTH-family peptides in a dose dependent manner. PTH-L and PTHrP were the most effective peptides in activating PTH1R (EC_50_ = 7.7 nM and EC_50_ = 22.7 nM, respectively). In contrast, PTH-L (100 nM) produced a small cAMP accumulation on activation of PTH3R but PTHrP and PTH (EC_50_ = 2.5 nM and EC_50_ = 22.1 nM, respectively) readily activated the receptor. PTHrP also stimulated intracellular Ca^2+^ accumulation on activation of PTH1R but not PTH3R.

**Conclusion:**

Two PTHR homologues of the vertebrate PTH1R and PTH3R were isolated and functionally characterized in chicken. Their distinct pattern of expression during embryo development and in adult tissues, together with their ligand preference, suggests that they have acquired specific functions, which have contributed to their maintenance in the genome. PTH2R and its activating ligand, TIP39, are absent from bird genomes. Nonetheless identification of putative PTH2R and TIP39 in the genome of an ancient agnathan, lamprey, suggests the PTH/PTHrP ligand and receptor family was already present in an early basal paraphyletic group of vertebrates and during the vertebrate radiation diverged via gene/genome duplication and deletion events. Knowledge of the role PTH/PTHrP system in early vertebrates will help to establish evolution of function.

## Background

The parathyroid hormone (PTH)-family consists of a group of structurally related factors that regulate calcium and bone homeostasis and a multitude of developmental processes (i.e. heart, mammary gland and immune system), which are mediated by calcium [1,2]. PTH, PTH-related protein (PTHrP) and the tuberoinfundibular peptide 39 (TIP39 a.k.a. PTH2) are members of the PTH-family in placental mammals. They are encoded by separate genes and in the protein share a conserved N-terminal amino acid sequence, which is involved in receptor binding and activation [3-5]. In non-mammalian tetrapods and fish, an additional family member designated PTH-L exists [2,6]. The specific whole genome duplication that occurred in teleost fish means they possess duplicated gene homologues of the mammalian forms of PTH (PTH1/PTH2) and PTHrP (PTHrPA/PTHrPB) [6-8]. Peptides of this family are proposed to have emerged early during the vertebrate radiation as suggested by the recent characterization of the PTH/PTHrP family members in the cartilaginous fish the elephant shark (*Callorhinchus milii*) [9]. The number of receptors (PTHRs) identified for PTH family ligands varies from two in mammals, designated PTH1R and PTH2R, to three in teleost fish, which do not appear to have the full complement of duplicated PTHRs but instead contain mammalian orthologues and a third receptor designated PTH3R [10]. Recently, putative PTHR were predicted also in the tunicate *Ciona intestinalis* and in the mollusc Antarctic clam *Laternula elliptica*, suggesting that the evolution of PTHRs may have predated the vertebrate radiation [11,12].

PTH/PTHrP receptors are members of the family 2 B1 G-protein coupled receptors (GPCRs), a large group of seven transmembrane peptide and neuroendocrine receptors characterised by the presence of a large N-terminal ectodomain (N-ted) involved in ligand interaction and by a C-terminal domain that is responsible for the activation of the intracellular signalling cascade [11,13-15]. At the N-ted, six conserved cysteine residues and N-glycosylation sites are responsible for the formation of the ligand-binding pocket. Receptor activation triggers different intracellular signalling pathways, including the activation of protein kinases A (PKA) and accumulation of cAMP [14,16,17] and phospholipase C leading to protein kinase C (PKC) and intracellular Ca^2+^ release [16,18]. Moreover, studies using *in vitro* cell assays and monitoring activation of intracellular signalling pathways established that receptor preference for the mammalian and teleost ligands are different. Human and zebrafish PTH1R are preferentially activated by both PTH and PTHrP. Zebrafish PTH2R binds exclusively to TIP39 while the human PTH2R is also activated by PTH [19]. PTH3R is preferentially activated by PTHrP, including fish PTHrPA [20-22]. Receptor preference for PTHrP1B and PTH-L remains to be determined.

Homologues of the mammalian PTH and PTHrP and the teleost PTH-L genes and transcripts were identified in chicken and *Xenopus*, and preliminary functional studies indicated that the N-terminal peptides are able to stimulate calcium transport [2]. As with the human gene, chicken PTHrP produces several distinct transcript isoforms, and has a widespread tissue distribution. Furthermore, PTH was highly expressed in the chicken parathyroid gland and was the most potent peptide promoting calcium transport across the chorionallantois membrane (CAM) [2]. Despite the current lack of knowledge about the function of the PTH/PTHR systems in chicken, putative PTHR activated by PTHrP were reported in chicken bone and kidney [23-25]. Studies describing the action of PTH and PTHrP on chicken tibial growth plate chondrocytes (GPCs) suggest that they activate identical intracellular signalling pathways to those described for the mammalian and teleost homologues [16,26]. Homologues of PTH1R and PTH3R are predicted to exist in the chicken genome whereas PTH2R and its specific peptide ligand TIP39 seem to be absent [27,28]. Moreover, based upon *in silico* analysis it has been hypothesised that chicken PTH1R may be a pseudogene [27].

The aim of the present study is to contribute for the understanding of the evolution and function of the PTH/PTHrP system in vertebrates by the isolation and characterisation of the PTH/PTHrP receptors in chicken. To this end, PTHR sequences were identified *in silico* in the chicken genome and expressed sequence tags (EST) deposited in public databases and the full-length receptors cloned. Gene expression in chicken adult and during embryonic development stages were characterised and the relative potency of the N-terminal (1–34) region of chicken PTH and PTHrP in stimulating receptor activation determined by quantification of intracellular cAMP production and Ca^2+^ accumulation. The origin and evolution of the PTH/PTHrP systems in the vertebrate radiation was revisited by comparison of chicken ligands and receptors with homologues in other metazoan genomes.

## Results

### The chicken PTH/PTHrP receptors

In the chicken genome putative PTH1R (ENSGALG00000005476) and PTH3R (ENSGALG00000019797) were identified. The predicted mature transcripts of chicken PTH1R and PTH3R are 1614 bp and 1626 bp in length that correspond to deduced proteins of 538 and 542 amino acids, respectively, both containing putative signal peptide sequences and sharing 54% overall amino acid sequence identity. The chicken PTH1R mRNA (Accession number FR746109, Additional file 1) was confirmed by RT-PCR on cDNA from whole chicken embryos at stage 26HH and from 3 ESTs found in database from stage 20-21HH whole chick embryos (BU219643), from stage 36HH limbs (BU401969) and from growth plate chondrocytes (BU419888). The chicken PTH3R (Accession number FR746110, Additional file 2) was also confirmed by RT-PCR on the same cDNA sample but no EST was found. Despite thorough database searches and attempts to amplify a chicken PTH2R receptor using degenerate primers on genomic DNA and cDNA a homologue was not identified.

**Figure 1:**
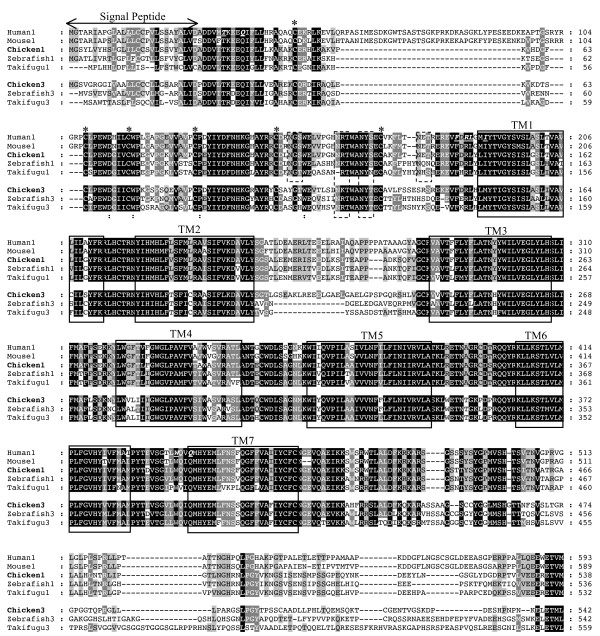
**Multiple sequence alignment of the chicken PTH1R and PTH3R with the mammalian and teleost homologues.** The predicted seven transmembrane domains (TM) are annotated and represented by boxes and the signal peptide sequence by an arrow. The six conserved cysteine residues are annotated with “*” and the conserved N-glycosylation consensus motif is dashed boxed. Amino acid residues involved in human with ligand-binding at the N-terminal and TM regions are in italics and underlined [13,31] and conserved motifs that were found to be determinant for PTH binding are annotated with “:” [11]. Accession numbers: Human1 (PTH1R, AAR1807), Mouse1 (PTH1R, NP_035329); Zebrafish1 (PTH1R, NP_571432); Zebrafish3 (PTH3R, NP_571453); Takifugu1 (PTH1R, CAD79707); Takifugu3 (PTH3R; CAD67555)

Multiple sequence alignment of the deduced amino acid sequence of chicken PTHRs with mammalian and teleost homologues (Figure 1) revealed the chicken PTH1R shares at least 76% similarity to other vertebrate PTH1Rs and that the chicken PTH3R is 71% similar with the zebrafish PTH3R. The seven transmembrane domains (TM) and the six cysteine residues at the N-ted are fully conserved across vertebrates (Figure 1) as are four putative N-glycosylation sites (Nx[TS]) in PTH1R two of which are also found in PTH3R. The chicken PTHR N-ted also contains the residues L^13^, T^33^, Q^37^, F^184^, R^186^, L^187^ and I^190^ previously identified to be involved in the interaction of mammalian PTH1R with PTH(1–34) and PTHrP(1–34) [13,29]. The only amino acid residues in N-ted that are not conserved are L^13^ which is replaced by I^13^ in PTH1R and I^190^ which substituted by M^190^ in PTH3R (Figure 1). The residues D^113^, W^118^, P^132^ and W^154^ which are involved in the structural conformation of the ligand-binding pocket of family 2 B1 GPCRs [11,30] are also conserved in the chicken receptor sequences.

### PTH/PTHrP receptor homologues in vertebrate and invertebrate genomes

To place in context the evolution of chicken PTHRs, potential PTH/PTHrP homologues from other vertebrate and invertebrate genomes were retrieved from public databases (Table 1). In *Xenopus*, and lizard genomes, partial sequences for putative PTH1R, PTH2R and PTH3R were identified. In the bird genomes - zebra finch, turkey and duck - only homologues of the chicken PTH1R (ENSTGUG00000000188, ENSMGAG00000002767 and ENSAPLG00000005598, respectively) and PTH3R (ENSTGUG00000001924, ENSMGAG00000001866 and ENSAPLG00000010124, respectively) genes were retrieved. The deduced mature peptide sequence of the bird PTHRs identified are highly conserved with the chicken homologues and share at least 75% and 83% with PTH1R and PTH3R, respectively. In the recently available genome of the cartilaginous fish, elephant shark, three potential receptors (homologues of the vertebrate PTH1R, PTH2R and PTH3R) were identified and in lamprey two PTH/PTHrP receptors seem to be present (Table 1). Among the invertebrate chordates two potential PTH/PTHrP receptors were identified in the *Ciona* genome (Ciona_a, ENSCING00000002669 on scaffold_67 and Ciona_b, ENSCING00000006282 in scaffold_162) and in the amphioxus *Branchiostoma floridae* genome (XM_002599399) a PTH/PTHrP receptor gene also appears to be present. Amino acid sequence similarity of the predicted Ciona_a and Ciona_b receptors is 46% and 49%, respectively, with the chicken PTH1R and 45% and 47% from amphioxus with the paralogue PTH3R. The deduced PTH receptor from amphioxus shares 49% and 47% sequence similarity with the chicken PTH1R and PTH3R, respectively.

**Table 1:** Accession numbers of predicted vertebrate PTHR genes

Common name	Scientific name	PTH1R	PTH2R	PTH3R
Human	*Homo sapiens*	ENSG00000160801	ENSG00000144407	*Not identified*
Opossum	*Monodelphis domestica*	ENSMODG00000013964	ENSMODG00000015872	*Not identified*
Platypus	*Ornithorhynchus anatinus*	ENSOANG00000020450	ENSOANG00000008008	*Not identified*
Chicken	*Gallus gallus*	ENSGALG00000005476	*Not identified*	ENSGALG00000019797
Zebra finch	*Taeniopygia guttata*	ENSTGUG00000000188	*Not identified*	ENSTGUG00000001924
Turkey	*Meleagris gallopavo*	ENSMGAG00000002767	*Not identified*	ENSMGAG00000001866
Duck	*Anas platyrhynchos*	ENSAPLG00000005598	*Not identified*	ENSAPLG00000010124
Lizard	*Anolis carolinensis*	ENSACAG00000004743	ENSACAG00000011092	ENSACAG00000003901
African clawed frog	*Xenopus tropicalis*	ENSXETG00000003683	ENSXETG00000008019	ENSXETG00000003243
Zebrafish	*Danio rerio*	ENSDARG00000020957	ENSDARG00000006678	ENSDARG00000018418
Takifugu	*Takifugu rubripes*	ENSTRUG00000013866	ENSTRUG00000006000	ENSTRUG00000002272
Elephant shark	*Callorhincus milii*	AAVX01252370.1 AAVX01556909.1 AAVX01305998.1	AAVX01159975.1 AAVX01100591.1	AAVX01012445.1 AAVX01295680.1
Lamprey	*Petromyzon marinus*	GENSCAN00000088236	GENSCAN00000077871	*Not identified*

Species names of published genomes and database accession numbers of identified PTH1R, PTH2R and PTH3R.

### PTH/PTHrP receptor gene structure and short-range gene linkage

The chicken PTH1R and PTH3R share a complex gene organisation and are composed of 13 exons and are identical with the predicted gene structures of their homologues in *Xenopus* and zebrafish (Additional file 1 and Additional file 2). This contrasts to human PTH1R that contains 15 exons within the mature receptor region. For both chicken PTH1R and PTH3R, the signal peptide region is encoded within the 1^st^ exon and the TM regions are distributed between the 5^th^ and 13^th^ exon.

**Figure 2:**
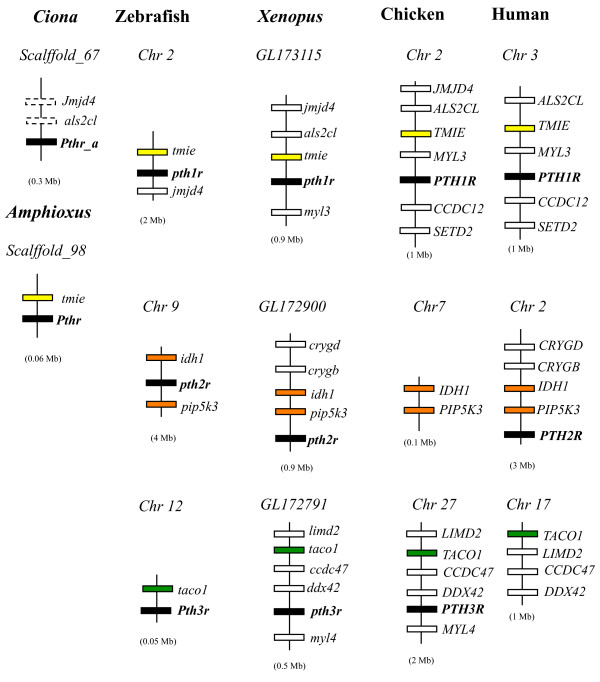
**Short-range gene linkage analysis of the chicken PTHRs with other metazoans.** The homologue genome regions in *Ciona* amphioxus, zebrafish, *Xenopus* and human were compared. Genes are represented by boxes and the size of the chromosome region analysed is given within brackets. Genes were named according to the HUGO annotation and lines indicate chromosome/scaffolds segments. The PTHR genes are represented by black boxes and gene name is in bold. The conserved linked genes are in colour to facilitate identification (yellow, PTH1R; orange, PTH2R and green, PTH3R) and an average of the distance compared is given within brackets. For simplicity, only syntenic genes are represented. Figure is not drawn to scale.

Gene synteny was maintained for both PTH1R and PTH3R and linked genes were found in chicken, zebrafish, *Xenopus* and human (Figure 2). The PTH1R gene maps to chicken chromosome 2, to human chromosome 3, to *Xenopus* scaffold 479, and to zebrafish chromosome 2 and the gene *TMIE* (transmembrane inner ear-like) was found to be conserved within the compared homologue genome regions. The chicken PTH3R gene maps to chromosome 27 and is homologous to *Xenopus* scaffold_155 and zebrafish chromosome 12. The gene *TACO1* (translational activator of mitochondrially encoded cytochrome c oxidase I) was found to be common in the teleost and tetrapod genome regions analysed.

Despite the apparent absence of a putative PTH2R gene, chicken homologue genes that are conserved in the vertebrate PTH2R gene environment were also found in a conserved cluster in the chicken genome (Figure 2). The genes *IDH1* (isocitrate dehydrogenase) and *PIP5K3* (1-phosphatidylinositol-4-phosphate 5-kinase) found in close proximity to the zebrafish, *Xenopus* and human PTH2R were identified on chicken chromosome 7. Similarly, the human genome homologues of the conserved vertebrate PTH3R gene environment were also identified on chromosome 17.

Comparative analysis with the homologue genome regions in the *Ciona* genome indicates that *Ciona* PTHR (Ciona_a) located in scaffold_67 shares gene environment conservation with the vertebrate PTH1R. Putative homologues of the vertebrate *ALS2CL* and *JMJD4* are also predicted in the *Ciona* homologue genome region, which contrasts with Ciona_b gene environment in scaffold_162, where there was no gene synteny. In the amphioxus genome a homologue of the vertebrate *TMIE* is predicted in close proximity with the putative PTHR locus (scaffold_98).

### Phylogenetic analysis of PTH/PTHrP receptors

**Figure 3:**
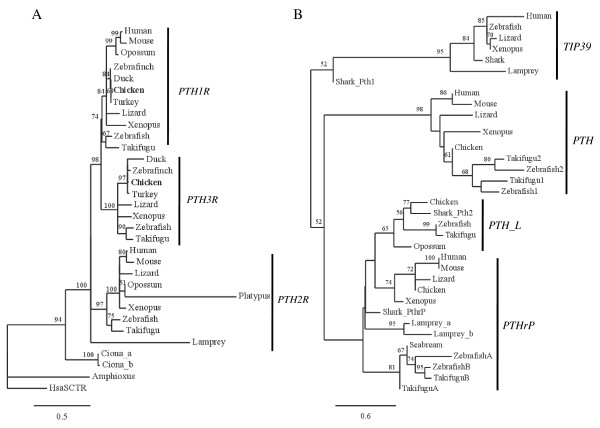
**Phylogenetic trees of (A) PTHRs and (B) PTH family peptides.** Consensus trees were constructed using Maximum Likelihood (ML) and 500 bootstrap replicates. Bootstrap values higher than 50% confidence are mapped. In **A**) rooted tree of the 31 taxa deduced mature protein sequences of the genes represented in Table 2 with human SCTR (HsaSCT) as outgroup. Chicken PTH1R and PTH3R are in bold and the *Platypus* PTH1R, the putative shark members and lamprey PTH1R were not utilized due to short predicted sequence. In **B**) unrooted tree of the deduced mature precursor sequence of PTH-family members from 35 taxa. Accession numbers: Human (PTH, AAH96144; PTHrP, AAA60216, TIP39 NP_848544); Mouse (PTH, NP_065648; PTHrP, CAC39218; TIP39 NP_444486); Chicken (PTH NM_205452, PTHrP NC_006092, PTH-L, CAW30790), Lizard (PTH ENSACAP00000018344; PTHrP ENSACAP00000018097); *Xenopus* (PTH FM955441, PTHrP NM_205338, TIP39 ENSXETP00000057310); Zebrafish (PTH1, NP_998115; PTH2, NP_998114; PTHrPA, AAY87956; PTHrPB, AAY87957; PTH-L, CU856139, TIP39 NP_991140); *Takifugu* (PTH1, CAG26460; PTH2, CAG26461; PTHrPA, CAB94712; PTHrPB, CAG26459); Seabream PTHrP AF197904_1; Elephant shark (Pth1 ADJ218, Pth2 ADJ21811, PthrP ADJ21812). Sequences from *Opossum* PTH_L (chromosome 2), Lizard TIP39 (GL343355.1), Elephant shark TIP39 (AAVX01305587.1), Lamprey Pth/PthrP (GL476611) and TIP39 (GL477315) were deduced from the genome sequence.

Phylogenetic analysis of the chicken PTH1R and PTH3R with the retrieved vertebrate and invertebrate homologues is shown in Figure 3A. The consensus tree obtained suggests that the PTH/PTHrP receptors emerged early during the deuterostome radiation. The vertebrate PTHR members shared a common ancestor gene with *Ciona* and amphioxus and have evolved via gene or genome duplications. Two major clades are present one containing the vertebrate PTH2R and the other clusters PTH1R and PTH3R. This suggests that after the initial gene duplication they have been under different evolutionary pressure and that the vertebrate PTH1R and PTH3R are the result of a recent duplication event. The chicken and other avian PTH/PTHrP receptors cluster within the PTH1R/PTH3R group, confirming their homology (Figure 3A). The three *Xenopus* and lizard PTH/PTHrP receptors group with the teleost homologues. In *Ciona* the existence of two PTHRs appears to be the result of a specific duplication event.

### TIP39 genes in vertebrates

The absence of PTH2R in chicken raises questions about the presence in the genome of its putative ligand TIP39. Searches performed on the chicken genome and EST databases failed to identify the homologue of human and teleost TIP39. However, a putative TIP39 gene is predicted in the *Xenopus* (ENSXETG00000027477) genome and also in non-annotated regions of the lizard (Scaffold GL343355), Platypus (SuperContig Contig8806) and Opossum (chromosome 4) genomes. In addition, in lamprey (GL477315) and elephant shark (AAVX01305587.1) a putative TIP39 gene also seems to be present. In elephant shark, four PTH-family genes were identified clustering with PTH, PTHrP, TIP39 and PTH-L (Figure 3B). No potential TIP39 gene was identified in the tunicate *Ciona* and amphioxus genomes, suggesting that this gene is specific to vertebrates. Phylogenetic analysis of the deduced TIP39 mature peptides with the PTH/PTHrP members confirmed that the TIP39 clade is basal and shared common origin (Figure 3B). Characterization of the vertebrate TIP39 gene environment revealed that gene order and gene synteny is only conserved in *Xenopus* and human and no conserved genes were found in teleost and lamprey (Additional file 3).

### Tissue gene expression

**Figure 4:**
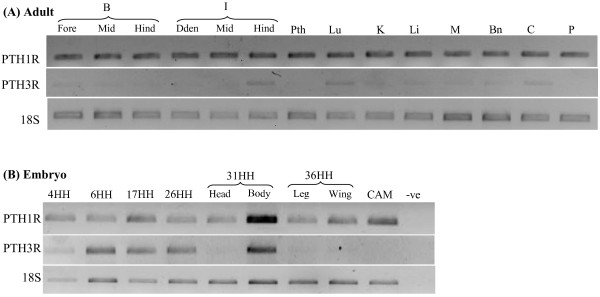
**Tissue expression of the chicken PTH1R and PTH3R in adult (A) and embryonic stages (B).** The RT-PCR reactions for **A**) and **B**) were carried out simultaneously using transcript specific primers and ribosomal subunit 18 S as reference. In B the chicken the embryos were at different Hamburger and Hamilton stages, (HH) and CAM was obtained from stage 44HH. B-brain (forebrain, midbrain, hindbrain); I-intestine; Dden-duodenum; Mid-midgut; Hind-hindgut; Pth-parathyroid; Lu-lung; K-kidney; Li-liver; M-muscle; Bn-bone; C-cartilage; P-pituitary; CAM- chorionallantois membrane; -ve-negative.

Tissue expression of the chicken PTHRs transcripts was characterised in several adult tissues and during embryo development. The chicken PTH1R was detected in all adult tissues and embryo stages analysed (Figure 4). During embryo development PTH3R was expressed from stage 4HH (19 hours of incubation) onwards, but was absent or down-regulated in the head of 31HH and 36HH. The chicken PTH3R was also detected in most of the adult tissues despite its low level of expression, but highest transcript expression was found in intestine (hindgut), lung, liver and cartilage (Figure 4).

### Receptor activation of cAMP production

**Figure 5:**
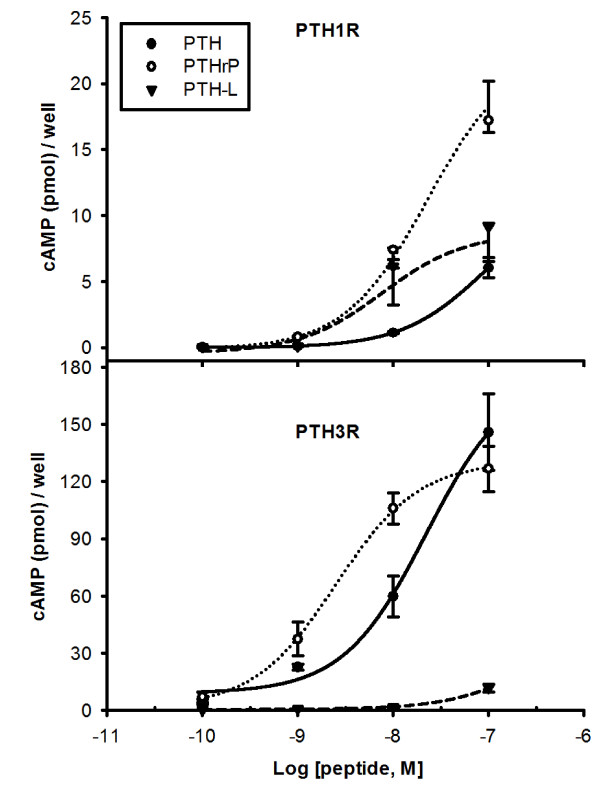
**Accumulation of cAMP in HEK293 cells transfected with chicken PTH1R and PTH3R.** Cells were stimulated with 100 nM to 0.1 nM concentrations of the truncated (1–34) chicken N-ted peptides PTH, PTHrP and PTH-L indicated with ○, ● and ▾, respectively. Values represent means ± SEM of three independent experiments performed in triplicate and read in duplicate.

The cAMP accumulation of a stable cell line expressing chicken PTH1R and PTH3R in the presence of chicken PTH(1–34), PTHrP(1–34) and PTH-L(1–34) is shown in Figure 5. All chicken peptides were able to activate the two receptors in a dose-dependent manner with different half maximal concentrations (EC_50_). Furthermore, the accumulation of cAMP in cells transfected with PTH3R was almost one order of magnitude larger compared to those with PTH1R. PTHrP produced the highest stimulation of PTH1R (p < 0.05) and together with PTH-L was the most efficient peptide (Table 2). Stimulation of PTH3R was highest for PTHrP and PTH, while PTH-L (100nM) only produced a small cAMP accumulation above basal levels and was approximately 13 times lower than PTH and PTHrP (p < 0.05). The EC_50_ followed the same pattern with PTH-L in the μM range (Table 2). Human PTH was also able to stimulate chicken PTH1R (21.4 ± 10 pmol/well at 10^-7^ M) (Additional file 4). Human TIP39 failed to induce accumulation of cAMP with PTH1R and only negligible cAMP production was obtained for PTH3R (Additional file 4).

**Table 2:** Potency and efficacy of the chicken 1–34 N-terminal PTH peptides on PTH1R and PTH3R cAMP production

	PTH1R			PTH3R		
	EC_50_ (nM)	95% Confidence Intervals (nM)	E_max_ (pmol/well)	EC_50_ (nM)	95% Confidence Intervals (nM)	E_max_ (pmol/well)
PTH	92.1	11.4 to 743.5	6.03 ± 0.78	22.1	7.1 to 68.4	144.7 ± 20.02
PTHrP	22.7	10.7 to 48.4	17.21 ± 1.84 *	2.5	1 to 6.1	119.9 ± 11.76
PTH-L	7.7	1.5 to 39.9	9.13 ± 1.44	306.9	27.7 to 3.4e^+10^	12.6 ± 2.13 *

Peptides tested, cAMP production, 95% confidence intervals and maximal cAMP production E_max_ (mean ± SEM). Each result is an average of 3 experiments carried out in triplicate. * indicates statistical significance compared to other peptides (p < 0.05).

### Receptor activation of intracellular Ca^2+^

**Figure 6:**
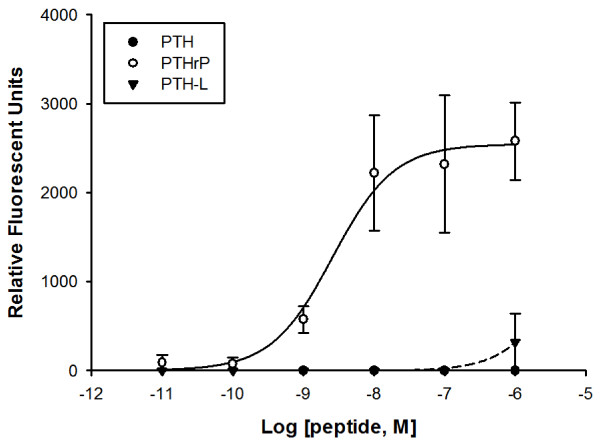
**Accumulation of iCa**^**2+**^**in HEK293 cells transfected with the chicken PTH1R.** Cells were stimulated with decreasing concentrations of the truncated (1–34) chicken peptides PTH, PTHrP and PTH-L indicated with ○, ● and ▾, respectively. Values represent means ± SEM from three independent experiments performed in triplicate.

A peptide screen using 1 μM and 100 nM of each PTH and PTHrP peptide revealed that irrespective of the peptide PTH3R activation did not cause release of intracellular Ca^2+^ (iCa^2+^). However, PTHrP efficiently stimulated iCa^2+^ accumulation with PTH1R with an EC_50_ of 2.6 nM and PTH-L (1 μM) caused only a slight stimulation and PTH had no effect (Figure 6).

## Discussion

Homologs of PTH1R and PTH3R, but not PTH2R, are present in the chicken genome, and are the result of a gene duplication, which occurred prior to the divergence of fish. The chicken PTHRs have a widespread tissue distribution and are activated by the N-terminal 1–34 fragment of PTH-family peptides. The presence in the two receptors of conserved amino acids important for ligand-binding in N-ted and residues W^437^ and Q^440^ located within the third extracellular loop and TM7, respectively, suggests the function of the chicken receptors is similar to the human PTH1R [13,32]. The genomic structure of chicken PTH1R is similar to fish and amphibian while the human homologue gene contains two extra exons: exon 2, which corresponds to an extracellular loop exclusive of the mammalian receptor and exon 15 which is the result of intron gain within one of the exons encoding the C-terminus of the receptor [33].

PTH1R and PTH3R are expressed during chicken development and in many different adult tissues, including the classical calcium-target tissues such as kidney, intestine, bone and cartilage. In human, PTH1R is also expressed in kidney and in a variety of other tissues, and is activated by both PTH and PTHrP, and accounts for the autocrine/paracrine function of PTHrP [19,34,35]. In fish, PTH1R was mainly expressed in the scales, liver, gonad, skin, brain and pituitary [1,36,37]. The *Xenopus* lung epithelium produces PTHrP which regulates, through a receptor-mediated mechanism, mesodermal leptin necessary for lung development [38]. The receptor responsible is likely to be PTH1R, which in *Xenopus laevis* is expressed in lung, brain, skin, kidney and bone [39]. There is much less information available about the characteristics of PTH3R and in chicken it is most highly expressed in the hindgut, lung and cartilage. In fish, PTH3R has been identified in the interrenal tissue where it may regulate cortisol secretion [40] and from intestine where it signals primarily via the adenylate cyclase/protein kinase A (AC/PKA) signalling pathway possibly mediating calcium uptake [41].

PTHRs are expressed in developing bone/cartilage structures in the embryo and have a key role during development. In mammals, PTH1R in association with Indian hedgehog (IHH) signalling regulate endochondral bone ossification and skeletal development [42-44]. Previous studies with chicken embryos reveal that, prior to the appearance of skeletal tissue, PTHrP and PTH1R are co-expressed by cells of the ectoderm, skeletal muscle, peripheral nerve and mesenchyme [25]. Hyaline cartilage in chicken first observed at HH27, co-express PTHrP and PTH1R in chondroblasts but by day 37 the chondrocytes do not express the receptor which is present in the perichondrium as well as in preosteoblasts, osteoblasts and osteocytes [25]. The calcium necessary for chicken embryo development is likely to be regulated by the egg CAM [2] and probably involves the activation of chicken PTH1R, which is the only PTHR amplified from this tissue. In the current study, the low expression of PTH3R in head, legs and limbs but high expression in the body of the developing embryo suggests this receptor may be more important in the development of non-skeletal tissues.

Chicken PTHRs are activated by PTH-family peptides in a dose-dependent manner. PTHrP(1–34) was the most potent peptide overall and stimulated cAMP accumulation through PTH1R and PTH3R and had overlapping potency with PTH(1–34) for the latter receptor. Mammalian PTH1R is activated by both PTH and PTHrP which have similar potency and stimulate both cAMP and iCa^2+^ production [45]. Zebrafish PTH1R is activated similarly by the N-terminal peptides of fugu PTHrPA, human PTHrP and PTH and zebrafish PTH1 but not zebrafish PTH2 while zebrafish PTH3R has preference for fugu PTHrPA, human PTHrP and zebrafish PTH1 and PTH2 but not for human PTH [8,10]. Human PTH activated chicken PTH1R and stimulated cAMP production with an apparently similar potency to chicken PTHrP. Interestingly, chicken PTH-L had a low capacity to activate any of the receptors, which is consistent with its lower potency in stimulating calcium transport across the CAM [2]. Whether PTH-L binds to another as yet unidentified receptor(s) or is a less active PTH-family member in chicken remains to be established.

Of the two chicken receptors, PTH3R accumulated one order of magnitude more cAMP than PTH1R. This is to some extent surprising as HEK293 cells express PTH1R at a low level [46]. It may indicate that the two receptors use a different complement of signalling molecules (e.g., G-protein α-subunits, adenylyl cyclases) or they may interact with receptor auxiliary proteins which remain to be characterised [46]. In contrast, only PTH1R stimulates accumulation of iCa^2+^. Similar observations were made for zebrafish PTH1R and PTH3R and activation with PTH/PTHrP peptides stimulated cAMP accumulation but only PTH1R, was capable of activating IP3/iCa^2+^ signalling [10]. In sea bream scales, where only PTH1R is expressed, piscine PTHrPA(1–34) activated both the adenylyl cyclase/protein kinase A (AC/PKA) and phospholipase C/protein kinase C (PLC/PKC) signalling cascades [37]. Deletions of N-terminal amino acids in piscine PTHrPA reduced cAMP accumulation, but had no effect on PLC/PKC signalling [37]. Moreover piscine PTHrP(1–34) was also found to stimulate cAMP accumulation in isolated sea bream enterocytes via PTH3R but no activation of the PLC/PKC pathway was detected [41]. The results of the present study indicate that chicken PTH1R and PTH3R are functionally different and this observation with the differing tissue expression supports the idea of differential cellular functions. It remains to be established if chicken PTHRs activate other intracellular signalling pathways such as the mitogen-activated protein kinase or interact with proteins such as RAMPs (receptor-activating-modifying proteins) or calmodulins known to modulate the activity of the mammalian receptor [reviewed by [19,47].

The functional importance of calcium from single cell organisms to metazoan has required the evolution of mechanisms for its regulation and the suggestion that the PTH family may be ancient. For example, it has been suggested that TIP39 may be present in yeast [48] but this seems to be the consequence of coincident gene names rather than authentic sequence similarity [49]. Immunohistochemical studies with heterologous antisera suggest that PTH exists in invertebrates such as snail, cockroach and amphioxus [50]. Studies in snail also suggest a potential neuropeptide role for the invertebrate PTH/PTHR system, as mammalian PTH was found to stimulate calcium influx in neurons and induce depolarization and modulation of neural transmission through the inositol-triphosphate second messenger system [50-52]. However, Western blot failed to detect PTH immunoreactive material in neural extracts of prawn, squid, cuttlefish, starfish, dogfish, skate or hagfish [52]. Moreover, mining of invertebrate molecular databases failed to identify putative PTH-family homologues and their existence remains to be conclusively demonstrated. In contrast, *in silico* analysis identified potential homologues of the vertebrate PTHRs in early deuterostome and protostome genomes [11,12].

**Figure 7:**
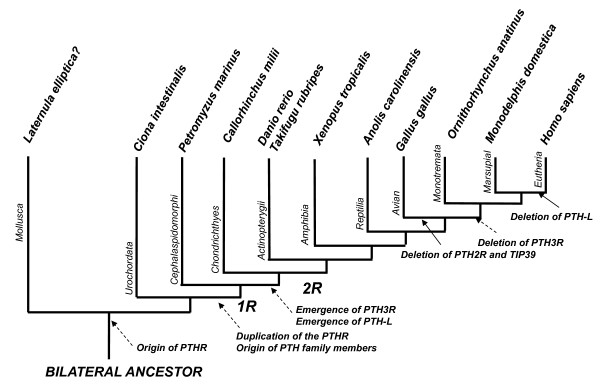
**Proposed evolutionary model of chordate PTH-family members and their receptors.** The genome events that are proposed to have influenced the vertebrate radiation (1R and 2R) are indicated. Dashed arrows indicate putative gene duplication events within the PTH/PTHrP system. “?” indicate gene not identified.

Both PTH/PTHrP and their receptors seem to be present in lamprey and cartilaginous fishes (Figure 7). In the lamprey, the genome region GL476611 contained two potential PTH/PTHrP-like genes that shared similarity with the vertebrate homologues and were in linkage with the *BTBD10* gene, which is present in the vertebrate PTH gene environment [2] (Additional file 3). A putative TIP39 gene is also present in the lamprey. In the elephant shark besides the previously reported *PTH* (*Pth1*) and *PthrP* homologues [9] putative TIP39 (*Pth2*) and *PTH-L* (*Pth2* of Liu et al. [9]) genes were identified. This suggests that the gene/genome duplication event that generated the vertebrate PTH/PTHrP family occurred prior to or early in the origin of vertebrates (Figure 7). However, isolation of full-length lamprey transcripts is still required to permit full characterization of the PTH/PTHrP family in early vertebrates.

Two putative PTHR-like receptor genes (PTH1R-like and PTH2R-like) and three homologues of the vertebrate PTH1R, PTH2R and PTH3R genes are predicted in the lamprey and elephant shark genomes, respectively, suggesting that the evolution of both peptides and their receptors occurred in the same time-frame. Nevertheless, the identification of putative PTHRs in invertebrate genomes and the apparent absence of homologues of their vertebrate PTH/PTHrP ligands suggest that PTHRs which occur in the invertebrate lineage pre-dated the emergence of their vertebrate ligands (Figure 7), an evolutionary pattern that is also common to other 2 B1 GPCR family members [53].

The duplication of PTH-family members and their specific receptors has occurred early in the vertebrate radiation. The emergence of seems to have accompanied the proposed two rounds of gene/genome duplication events that occurred at the basis of vertebrates [54-57]. The putative PTH1R/PTH3R gene precursor and PTH2R gene are suggested to have emerged earlier, prior to or at the origin of vertebrates (1R), and PTH1R and PTH3R genes diverged subsequently prior to the *Chondrichthyes* (cartilaginous fish) radiation (2R). If a putative duplicate PTH4R gene existed it was eliminated from the genome.

The origin of PTH/PTHrP family members and TIP39 occurred early in vertebrate evolution and probably before the divergence of lamprey since a putative PTH-L was found in cartilaginous fish. Later in evolution gene deletions occurred as revealed by the absence of PTH2R and its ligand TIP39 in birds, proposed to result from a chromosome segment deletion in the bird genome [28]. Similarly, PTH3R and PTH-L are absent from placental mammal genomes (human and mouse). Thus, while putative ligands are only discernible in early vertebrates, the receptors seem to have an earlier origin.

## Conclusions

Two PTHR homologues of the vertebrate PTH1R and PTH3R were isolated and functionally characterized in chicken. Their distinct pattern of expression during embryo development and in adult tissues, together with their distinct activation response to the potential ligands, suggests that they have acquired specific functions, which contributed to their maintenance in the bird genome. The chicken genome lacked a homologue of the vertebrate PTH2R and of its specific ligand TIP39, a feature shared by all avian genomes analysed. PTHR and PTH-family members have evolved via gene/genome duplications and deletion events and the identification of putative homologues in the primitive lamprey genome suggests that they emerged early and co-evolved during the vertebrate radiation. Knowledge of the role PTH/PTHrP system in early vertebrates will help to establish their functional evolution.

## Methods

### Animals and tissue collection

All procedures with animals were performed in accordance with Portuguese legislation under a “Group-1” licence from the Direcção-Geral de Veterinária, Ministério da Agricultura, do Desenvolvimento Rural e das Pescas, Portugal. Tissues for gene expression analysis were obtained from adult white leghorn chickens (*Gallus gallus*), anesthetized with diethyl ether (Merck, Spain) before sacrifice by decapitation. Fertile chicken eggs to produce embryos were obtained from Quinta da Freiria (Serpa, Portugal) and kept at 37.6°C under high humidity in an automatic incubator (Brinseca OCTAGON 40) with gentle rotation. Developmental stages [58] selected for analysis coincided with the presence of prominent calcified structures: 4 HH (definitive primitive streak process); 6 HH (head and neural folds); 17 HH (leg bud formation); 26 HH (toes formation); 31 HH (feather germs; emergence of the interdigital membrane) and 36 HH (labial groove; uropygial gland). The chorionallantois membrane (CAM) from 44 HH which increases calcium transport in response to PTH-family peptides [2] was also collected. Tissues were snap frozen in liquid nitrogen and stored at −80°C until use.

### In silico database searches

Genomes and EST databases were interrogated for PTH/PTHrP receptor homologues using the human (PTH1R, AAR18076; PTH2R, AAH36811) and zebrafish (PTH1R, NP_571432; PTH2R, NP_571452; PTH3R, NP_571453) deduced protein sequences with tBLASTn and default settings [59]. The following databases were searched: 1) the Ensembl genome and pre_ensembl (http://www.ensembl.org) genome assemblies of chicken, opossum (*Monodelphis domestica*), platypus (*Ornithorhynchus anatinus*), zebra finch (*Taeniopygia guttata*), turkey (*Meleagris gallopavo*), duck (*Anas platyrhynchos*), toad (*Xenopus tropicalis*), lizard (*Anolis carolinensis*), sea lamprey (*Petromyzon marinus*), 2) the the elephant shark (*Callorhinchus milii*) genome assembly at http://esharkgenome.imcb.a-star.edu.sg, 3) the chicken EST database (dbEST) of the Biotechnology and Biological Sciences Research Council (http://www.chick.manchester.ac.uk/), the dbEST subsets Aves (taxid:8782), lamprey (taxid:7745) and cartilaginous fishes (taxid:7777) of the National Center for Biotechnology Information (NCBI, http://www.ncbi.nlm.nih.gov/). Due to the short size of contigs of the shark genome assembly searches were carried out using the coding sequence of the human and zebrafish PTHRs transmembrane (TM) domains retrieved from PRINTs database (http://www.bioinf.manchester.ac.uk/dbbrowser/PRINTS/).

The presence of a putative TIP39 and PTH/PTHrP genes in early vertebrate genomes of the elephant shark, lamprey and early deuterostomes *Ciona intestinalis* and *Branchiostoma floridae* (JGI genome assembly, http://www.jgi.doe.gov/) and in protostomes were also investigated using a similar search strategy using the human (Q96A98) and zebrafish (AAI64665) TIP39 sequences and the Fugu PTHrPA (Q9I8E9), PTHrPB (CAG26459), PTH1 (CAG26460) and PTH2 (CAG26461) and PTH-L (CAG26462) sequences.

### Sequence annotations and comparative analysis

The deduced mature peptide sequences were obtained from BCM search launcher (http://searchlauncher.bcm.tmc.edu/seq-util) and the localisation of the putative signal peptide region predicted using SignalP (http://www.cbs.dtu.dk/services/SignalP). TM domain regions were deduced using TMHMM (http://www.cbs.dtu.dk/services/TMHMM) and were manually edited according to the PRINTS annotation (http://www.bioinf.manchester.ac.uk/dbbrowser/PRINTS/). Multiple sequence alignments of the amino acid sequence of chicken PTH/PTHrP receptors were performed using ClustalX [version 1.83 [60]] with the following parameters: Gonnet series matrix, Gap opening penalty 10, Gap extension 0.2. The alignments were displayed and manually edited and percentages of sequence similarity and identity calculated using GeneDoc [61]. Phylogenetic trees were constructed using the deduced amino acid sequences of the full-length receptor and were built with the maximum likelihood (PhyML 3.0) [62,63] and Distance methods using Neighbour Joining (BioNJ and Neighbour) [64]. A substitution JTT model gave the best fit for the protein dataset in PROTTEST and was used for phylogenetic tree construction [65]. Maximum likelihood analysis was performed with 500 bootstrap replicates with a discrete gamma distribution of rates among sites with 4 categories. Distance methods performed 1000 bootstrap replicates and ProtDist [66]. Both maximum likelihood (ML) and distance methods generated similar tree topologies. The human secretin receptor (HsaSCTR, AAA64949) was used as an out-group.

An unrooted phylogenetic tree of PTH-family members was also carried out using the deduced mature protein of the vertebrate genes. Consensus trees were constructed following a similar strategy to that described for the PTHRs and both maximum likelihood and distance methods generated similar tree topologies.

### Gene structure and gene linkage analysis

Characterization of the chicken PTH/PTHrP receptor gene structures was performed on the basis of Ensembl gene predictions and by searching the genome with the nucleotide sequences of the mature receptor precursor using the NCBI Spidey interface (http://www.ncbi.nlm.nih.gov/spidey/). Intron-exon boundary splice sites (AG/GT) were manually confirmed. The immediate gene environment of the chicken PTHRs was characterised using the NCBI genome chromosome annotations and compared with the homologue genome regions in Human, *Xenopus* and zebrafish and also with *Ciona*. The human and zebrafish gene environment was accessed via the NCBI Mapview (http://www.ncbi.nlm.nih.gov/mapview/) and the *Xenopus* and *Ciona* retrieved from Ensembl. Genes and gene order flanking PTHRs in the zebrafish and human genomes were confirmed using the Vertebrate Genome Annotation (VEGA) database (http://vega.sanger.ac.uk/). The gene environment of vertebrate TIP39 and PTH was also compared in lamprey, zebrafish, *Xenopus* and human using a similar strategy.

### Analysis of gene expression

The methodology for production of cDNA from reverse transcription of total RNA and polymerase chain reaction (PCR) methodologies have been described by Pinheiro et al. [2]. The PTH1R PCR used primers PTH1Rfw atgggatcatatctggtttat and PTH1Rrv ggccagcagacaatacca with denaturation at 94°C for 3 min, 30 cycles of 94°C 30 sec; 55°C 30 sec; 72°C 2 min and a final elongation step at 72°C for 10 min. PTH3R was amplified using the primers PTH3Rfw atggggtctgtgggcagg and PTH3Rrv gttgaagtcgtagatgtagtc and 35 cycles with annealing at 57°C. Since genome searches were negative for PTH2R and to confirm it was not a genome sequence gap, primers PTH2Rfw_1_ caaagtagttcatacacatataggagt, PTH2Rfw_2_ tgcctcacacatttactgg and PTH2Rrv ggactggctgctggtgct were designed using the conserved regions of known vertebrate sequences and PCR reactions were performed as for PTH1R using the same panel of tissues. The ribosomal subunit 18 S was used as reference gene with primers 18Sfw tcaagaacgaaagtcggagg and 18Srv ggacatctaagggcatcaca and 22 thermocycles in the same conditions as for PTH1R. The PCR products were analysed on 1.5% agarose gel and products sequenced to confirm identity.

### Expression vector constructs

The complete coding regions, including the stop codon, of the chicken PTH1R and PTH3R were amplified from embryo limbs (36 HH) cDNA with the primers PTH1Rfw atgggatcatatctggtttat and PTH1Rfinalrv ttacatcactgtctctctttc for PTH1R and PTH3Rfw atggggtctgtgggcagg and PTH3Rfinalrv tcatagcatcgtctccagct for PTH3R. Taq DNA proofreading polymerase (Advantage® 2, polymerase mix, Clontech, France) was used in the PCR reactions according to the manufacturer’s instructions with 10X Advantage 2 PCR Buffer, 0.2 mM dNTP’s (GE Healthcare, Spain) and 0.25 μM of each specific primer for a final volume of 25 μl. The PCR reaction was carried out using the following thermocycle, 94°C 2 min, 30 cycles of 94°C 30 sec, 57°C for PTH1R and 59°C for PTH3R for 1 min, 72°C for 2 min, followed by 72°C for 10 min. The PCR products obtained were gel extracted using a GFX -PCR DNA and Gel Band Purification kit (Amersham Biosciences, Spain) and cloned into a pcDNA3.1/V5-His-TOPO expression vector (Invitrogen, USA) according to the manufacturer’s instructions. The bacterial clones obtained were PCR screened using vector and receptor specific primers and colonies that contained the coding regions in frame with the promoter vector pCMV were selected and plasmid DNA was extracted using the DNA Midi-Prep kit (Roche, Germany). To facilitate receptor integration in the genome, approximately 5 μg of the recombinant vectors were linearized with the restriction enzymes *ApaI* for PTH1R and *BglII* for PTH3R (Promega, Spain). The digested products were purified using phenol:clorophorm, ressuspended in 20 μl of distilled DNAse-free water and 5 μl was used to double transfect human embryonic kidney cell line 293 (HEK293 from European Collection of Cell Cultures; Salisbury, UK).

### Cell transfection and receptor stable cell line production

HEK293 cells were maintained in complete Dulbecco’s modified Eagle’s medium (DMEM, Sigma, Spain) with 4.5 g/L glucose, 110 mg/L sodium pyruvate and L-glutamine supplemented with 10% sterile foetal bovine serum and 0.1% penicillin: streptomycin antibiotic mixture (10.000 U:10 mg/ml, Sigma) in a humid 5% CO_2_ incubator (Sanyo) at 37°C. On the day prior to transfection, 2–3 x10^5^ cells were seeded on 6 well-plates (Starsted, Portugal). Linearized constructs (1 μg) and Fugene 6 (Roche, Germany) cell transfection reagent were used to transfect cells according to the manufactures protocol. The transfection complex was incubated for 40 min at room temperature before adding to the cells which were left to grown for 72 hours with daily changes of complete medium. Selection of the stably transfected clones was performed with complete medium supplemented with 800 μg/ml of Geneticin (G418 sulphate, GibcoBRL) and 250 μg/ml sterile filtered 1:100 amphotericin B solution (Sigma, Spain). Cell recovery was monitored daily by constant changes of antibiotic selective medium and the success of gene integration and expression was confirmed by RT-PCR with receptor specific primers.

### Receptor activation of cAMP

The capacity of chicken (1–34) PTH-family peptides to activate the stably transfected PTH1R and PTH3R cell lines was analysed by measuring intracellular cAMP production. Cell line bearing chicken receptors PTH1R and PTH3R were assayed in triplicate in the same assay in 96 well plates (Starstedt). Three independent experiments were performed. Cells were incubated at 37°C in a CO_2_ incubator and two days prior to the experiments, 2–3 x 10^5^ cells were plated and peptide assays were carried out by adding to the recombinant cells decreasing concentrations of test peptide: 100 nM to 0.1 nM of chicken PTH(1–34), PTHrP(1–34) and PTH-L(1–34) or 100 nM of human PTH(1–34) and human TIP39 (Sigma, Spain). Prior to peptide addition, cells were incubated for 40 min with 1 mM 3-isobutyl-1-methylxantine (IBMX, Sigma), which was replaced with fresh medium containing the test peptide in the presence of 1 mM IBMX for a further 40 min. Forskolin (10 μM, Sigma) was used as positive control. Negative control assays were carried out using non-transfected cells in the presence or absence of 100 nM of each peptide. At the end of the assays, cells were washed, ressuspended in 100 μl of 1 x PBS/0.5 M EDTA and immediately frozen at −80°C to promote cell lysis for later quantification of intracellular cAMP production.

For quantification of intracellular cAMP, cells were lysed using 3 consecutive thaw (42°C)/freeze (−80°C) cycles and the supernatant and cell debris were sonicated for 20 sec on ice. Cells were boiled at 100°C for 10 min to denature proteins and the supernatant was collected after centrifugation for 10 min at 4°C and 19000 g. cAMP was quantified in duplicate by radioimmunoassay using the TRK432 kit (GE Healthcare, UK) following the manufacturer's instructions. Data was normalized by subtracting basal cAMP accumulation in the negative controls and production above basal levels per well (cAMP/well) was plotted against peptide concentration. cAMP was also quantified in HEK293 cells stably transfected with the pCMV-GFP (vector expressing green fluorescent protein) to confirm receptor expression and the results for cAMP accumulation were equivalent to the peptide assay negative controls indicating that the vector construct in the cells did not interfere with cAMP production.

### Receptor activation of intracellular Ca^2+^

Intracellular Ca^2+^ (iCa^2+^) release was measured using the Ca^2+^ sensitive fluorescent dye Fluo-4 NW (Molecular Probes, Invitrogen) according to manufacturer’s instructions. Prior to the assay, plates (96 well black/plates, μClear bottom, Greiner, Germany) were treated with sterile poly-L-lysine (0.1 mg/ml, Sigma, Spain) to avoid cell release. Approximately 1 x 10^5^ cells in 100 μl of selective medium were plated per well and allowed to attach for 2 days. Medium was removed and cells were washed once with 2.5 mM probenecid (Molecular Probes, Invitrogen, Spain) in 1 x PBS and incubated for 30 min at 37°C with 100 μl of the Fluo-4 NW dye followed by an additional 30 min incubation period at room temperature. Incubations were carried out with 100 μl of the different peptides concentrations (from 1 μM to 100 nM) and cell fluorescence was measured every 10 sec over 2 minutes after addition of reagent using a Synergy4 (Biotek, USA) plate reader. Carbachol (100 nM; Sigma, Spain) was used as a positive control for the assay and the background signal was determined by measuring fluorescence in wells containing non-transfected cells. An assay negative control was carried out using non-transfected cells incubated with 100 nM peptide. Data was analysed by subtracting the background fluorescent values and plotted against peptide concentration.

### Statistics

The results are presented as the mean ± SEM of three independent experiments carried out in triplicate for cAMP experiments and two independent experiments carried out in quadruplicate for Ca^2+^ experiments. Data was plotted as the output of cAMP or Ca^2+^ at different peptide concentrations for each PTHR using SigmaPlot 9 (Systat, Inc., San Jose, CA). EC_50_ and confidence limits were calculated using a sigmoidal curve fitting feature within the Single Ligand Binding routine of the Pharmacology module of Sigmaplot. Unfortunately, the commercial radioimmunoassay for cAMP was withdrawn from the market while the study was already advanced and it was not possible to find an equivalent assay for tests at higher peptide concentrations to ensure saturation was achieved for all peptides. To improve curve fitting the maximum empirical stimulation achieved for each receptor was assumed to be at 100 nM for all assays and the curve fitted accordingly.

## Competing interests

The authors declare no financial competing interests.

## Authors’ contributions

PLCP did the experimental work on chicken (database searches, cDNA isolation, PCR), analyzed results and wrote the initial draft of the manuscript; JCRC supervised the comparative and phylogenetic analysis, carried out the receptor functional analysis, and contributed to the writing; DMP contributed to the planning of the work, analysis of results and writing; AVMC devised the work, obtained funds, analysed results and contributed to the writing of the manuscript. All authors read and approved the final manuscript.

## Supplementary Material

Additional file 1 **Nucleotide and deduced amino acid sequence of the chicken PTH1R.** Deduced sequence for PTH1R is based upon EST data and PCR amplification. Primer localization is represented by horizontal arrows and the exons change by vertical arrows. The TM domains are represented by bound lines and signal peptide in italic and bold. Cysteine residues are circled and putative N-glycosylation sites are boxed. Click here for file

Additional file 2 **Nucleotide and deduced amino acid sequence of the chicken PTH3R.** Sequence deduced based upon EST data and PCR amplification. Primer localization is represented by horizontal arrows and the exons change by vertical arrows. The TM domains are represented by bound lines and signal peptide by in italic and bold. Cysteine residues are circles and putative N-glycosylation sites are boxed. Click here for file

Additional file 3 **Comparison of the vertebrate TIP39 and PTH gene environments.** Only homologue genes are represented with the exception of the zebrafish TIP39 gene environment to demonstrate the lack of gene synteny with other vertebrate homologue regions. Dashed boxes represent putative gene locus in lamprey. Click here for file

Additional file 4 **Accumulation of cAMP in HEK293 cells transfected with chicken PTH1R and PTH3R.** Human PTH and TIP39 peptides were used at 10 nM and 100 nM. Values represent means ± SEM of a single experiment carried out in triplicate. Click here for file
