# Supplementary material for: Functional characterization and evolution of PTH/PTHrP receptors: insights from the chicken
Source: BMC Evol Biol. 2012 Jul 6;12:110. doi: 10.1186/1471-2148-12-110 (PMC3483286; doi:10.1186/1471-2148-12-110)
Supplement: Additional file 1 — Nucleotide and deduced amino acid sequence of the chicken PTH1R. Deduced sequence for PTH1R is based upon EST data and PCR amplification. Primer localization is represented by horizontal arrows and the exons change by vertical arrows. The TM domains are represented by bound lines and signal peptide in italic and bold. Cysteine residues are circled and putative N-glycosylation sites are boxed. [file 1471-2148-12-110-S1.pdf]

Additional file 1

ttctggagagtgaggtggagggggggggaggttggagggagggaaggagaaatcatctgcaagcggag 68
cgctggaagacctctccgtcctccaacttccccgagagagcgtggatgctggaaggagccccttggat 136

PTH1Rfw

ATG GGA TCA TAT CTG GTT TAT CAC AGC CTG GGT TTG ATC CTC TGC TGC TCC 187
M G S Y L V Y H S L G L I L C C S 17

GTG CTG AGC TCG GTC TAC GCT CTG GTG GAT GCT GAT GAT GTC ATC ACC AAA 238
V L S S V Y A L V D A D D V I T K 34

GAG GAG CAG ATC TTT CTA CTG CTA AAA GCC AAG GCC AAG TGC GAG CGA CAC 289
E E Q I F L L L K A K A K C E R H 51

CTG AAA GCC AAG GTG CCC AAG GTG CAT GAT GGC TTT TGT CTT CCT GAG TGG 340
L K A K V P K V H D G F C L P E W 68

PTH1Rrv

GAT GGT ATT GTC TGC TGG CCA GAA GGT GTG CCG GGC AAA GTG GTG GCT ATG 391
D G I V C W P E G V P G K V V A M 85

CCA TGT CCT GAG TAC ATC TAT GAC TTC AAT CAC AAA GGC CAT GCT TAT CGC 442
P C P E Y I Y D F N H K G H A Y R 102

CGG TGT GAC CTG AAT GGA AGC TGG GAG CTG GTT CCA GGC AAC AAC CGC ACC 493
R C D L N G S W E L V P G N N R T 119

TGG GCA AAC TAC AGT GAA TGT GCC AAG TTC CTC ACC AAC GAG ACA AGG GAG 544
W A N Y S E C A K F L T N E T R E 136

AGG GAG GTC TTT GAT CGC CTC TAT TTG ATT TAT ACT GTT GGA TAC TCC ATC 595
R E V F D R L Y L I Y T V G Y S I 153

TM1

TCT CTG GGA TCC CTC ACA GTT GCT GTC CTT ATC CTG GGA TAC TTC AGG CGT 646
S L G S L T V A V L I L G Y F R R 170

TTG CAC TGC ACT AGA AAC TAC ATC CAC ATG CAC CTG TTT GTC TCC TTC ATG 697
L H C T R N Y I H M H L F V S F M 187

TM2

TTG AGA GCT GTG AGC ATC TTC GTG AAG GAC GCG GTC TTA TAC TCT GGG TCA 748
L R A V S I F V K D A V L Y S G S 204

GCT TTG GAG GAG ATG GAG CGG ATT TCT GAG GAA GAC CTG AAA TCC ATA ACT 799
A L E E M E R I S E E D L K S I T 221

GAA GCA CCT CCA GCA GAT AAG TCA CAG TTT GTG GGT TGC AAA GTA GCG GTT 850
E A P P A D K S Q F V G C K V A V 238

ACC TTC TTC CTC TAC TTC CTG GCA ACC AAT TAC TAC TGG ATT CTG GTG GAA 901
T F F L Y F L A T N Y Y W I L V E 255

TM3

GGG CTC TAT CTC CAC AGC CTC ATC TTC ATG GCT TTT TTC TCA GAG AAG AAG 952
G L Y L H S L I F M A F F S E K K 272

TAT CTT TGG GGA TTC ACA TTA TTT GGC TGG GGA CTC CCT GCT GTA TTT GTT 1003
Y L W G F T L F G W G L P A V F V 289

TM4

ACA GCG TGG GCC AGC GTG AGA GCC ACT CTA GCT GAC ACA GAG TGT TGG GAC 1054
T A W A S V R A T L A D T E C W D 306

TTG AGT GCT GGC AAT TTA AAA TGG ATT ATT CAG GTG CCC ATC CTG GCA GCT 1105
L S A G N L K W I I Q V P I L A A 323

TM5

```
ATC GTG GTA AAT TTT ATT CTT TTT ATC AAT ATT ATC AGA GTC CTA GCA ACC  1156
 I   V   V   N   F   I   L   F   I   N   I   I   R   V   L   A   T   340

AAG CTA CGG GAA ACA AAT GCA GGG AGG TGT GAC TCA CGA CAA CAG TAC AGG  1207
 K   L   R   E   T   N   A   G   R   C   D   S   R   Q   Q   Y   R   357

AAG CTG CTG AAA TCT ACC CTC GTC CTT ATG CCT CTG TTT GGC GTT CAC TAT  1258
 K   L   L   K   S   T   L   V   L   M   P   L   F   G   V   H   Y   374
                        TM6
ATT GTT TTC ATG GCT ATG CCA TAC ACA GAT GTG TCA GGG ATT CTT TGG CAA  1309
 I   V   F   M   A   M   P   Y   T   D   V   S   G   I   L   W   Q   391

GTT CAA ATG CAC TAT GAA ATG CTG TTC AAC TCT TTC CAG GGA TTT TTT GTT  1360
 V   Q   M   H   Y   E   M   L   F   N   S   F   Q   G   F   F   V   408
                                     TM7
GCC ATC ATA TAC TGT TTT TGC AAT GGA GAG GTC CAA GCA GAA ATA AAG AAG  1411
 A   I   I   Y   C   F   C   N   G   E   V   Q   A   E   I   K   K   425

TCA TGG AGC AGG TGG ACA TTA GCA CTT GAT TTT AAA AGG AAA GCA CGA AGT  1462
 S   W   S   R   W   T   L   A   L   D   F   K   R   K   A   R   S   442

GGG AGC ACA ACC TAC AGT TAT GGA CCA ATG GTT TCC CAC ACC AGC ATC ACA  1513
 G   S   T   T   Y   S   Y   G   P   M   V   S   H   T   S   I   T   459

AAT GTA GCC ACG AGA GGG GCA CTT GCC CTC CAT CTC AAT ACA AGA CTT ATA  1564
 N   V   A   T   R   G   A   L   A   L   H   L   N   T   R   L   I   476

CCA GGG ACC CTC AAT GGA CAC CGG AAT TTA CCA GGT TAT GTA AAA AAC GGC  1615
 P   G   T   L   N   G   H   R   N   L   P   G   Y   V   K   N   G   493

TCT ATT TCT GAA AAC TCT ATG CCT TCT TCT GGA CCA GAG CAG TAC AAC AAA  1666
 S   I   S   E   N   S   M   P   S   S   G   P   E   Q   Y   N   K   510

GAT GAG GAG TAC CTG AAT GGC TCT GGG CTT TAT GAT GGA GAC AGA CCC ACA  1717
 D   E   E   Y   L   N   G   S   G   L   Y   D   G   D   R   P   T   527
                            PTH1Rfinalrv
GTA CTT GTT GAA GAA GAA AGA GAG ACA GTG ATG TAA                      1750
 V   L   V   E   E   E   R   E   T   V   M   *                       538
```
