# Supplementary material for: Functional characterization and evolution of PTH/PTHrP receptors: insights from the chicken
Source: BMC Evol Biol. 2012 Jul 6;12:110. doi: 10.1186/1471-2148-12-110 (PMC3483286; doi:10.1186/1471-2148-12-110)
Supplement: Additional file 2 — Nucleotide and deduced amino acid sequence of the chicken PTH3R. Sequence deduced based upon EST data and PCR amplification. Primer localization is represented by horizontal arrows and the exons change by vertical arrows. The TM domains are represented by bound lines and signal peptide by in italic and bold. Cysteine residues are circles and putative N-glycosylation sites are boxed. [file 1471-2148-12-110-S2.pdf]

Additional file 2

```
PTH3Rfw
ATG GGG TCT GTG GGC AGG GGA GGC ATC GTG GCT GCT CTG CTC TGC TGC TGC  51
 M   G   S   V   G   R   G   G   I   V   A   A   L   L   C   C   C   17

CTG CTG GGC TCT GCC CGG GCT CTG GTG GAT CCT GAC GAT GTT CTC ACT AAG  102
 L   L   G   S   A   R   A   L   V   D   P   D   D   V   L   T   K   34

GAA GAG CAG ATT TAC CTC CTG GTG GAA GCC AGA GAG AAA TGT CAG AGA GAC  153
 E   E   Q   I   Y   L   L   V   E   A   R   E   K   C   Q   R   D   51

ATC AGA GCT CAG CTG GAG AAG GTC AAA GAC ACC AGC TGC CTC CCA GAA TGG  204
 I   R   A   Q   L   E   K   V   K   D   T   S   C   L   P   E   W   68

GAT GGG ATC ATT TGC TGG CCC AAA GGC TCT CCC AGC CAG GAG GTG GCC GTG  255
 D   G   I   I   C   W   P   K   G   S   P   S   Q   E   V   A   V   85
                        PTH3Rrv
CCC TGC CCT GAC TAC ATC TAC GAC TTC AAC CAT AAA GGC CGT GCC TAC AGG  306
 P   C   P   D   Y   I   Y   D   F   N   H   K   G   R   A   Y   R   102

TAC TGC AGT GCC TAC GGG ACC TGG GAA GTG ACC CTC AGC CTC AAC AAG ACC  357
 Y   C   S   A   Y   G   T   W   E   V   T   L   S   L   N   K   T   119

TGG GCC AAT TAC ACT GAA TGC GCT GTG CTC TTC TCC TCC GAG AGC CGG AGC  408
 W   A   N   Y   T   E   C   A   V   L   F   S   S   E   S   R   S   137

CGT GAG AAG GAG GTG TTT GAC CGC CTG CAC TTG ATG TAC ACC ATT GGC TAC  459
 R   E   K   E   V   F   D   R   L   H   L   M   Y   T   I   G   Y   153

TCC ATC TCC TTG GCC TCC CTC ATC GTA GCT GTC TGC ATC CTC TCC TAC TTC  510
 S   I   S   L   A   S   L   I   V   A   V   C   I   L   S   Y   F   170
                                 TM1
AAG CGC CTG CAC TGC ACT CGC AAC TAC ATC CAC GTG CAC CTC TTC ACC TCC  561
 K   R   L   H   C   T   R   N   Y   I   H   V   H   L   F   T   S   187
                                                 TM2
TTC ATC TGC CGG GCG GCG AGC ATC TTC CTG AAG GAC GCG GTG CTC TAC TCG  612
 F   I   C   R   A   A   S   I   F   L   K   D   A   V   L   Y   S   204

GGC ACG CTG GGC AGC GAG GCG AAG CTG CGG GAG GAG GAG CTG GGG GCA GAG  663
 G   T   L   G   S   E   A   K   L   R   E   E   E   L   G   A   E   221

CTG GGG GCA GAG CTG GGC CCC TCG CCG GGC CAA CGC AGC CAC CTG GTT GGC  714
 L   G   A   E   L   G   P   S   P   G   Q   R   S   H   L   V   G   238

TGC AAG GTG GTG GTG ACG CTC TTC CTC TAC TTC TTG GCC ACC AAC CAC TAC  765
 C   K   V   V   V   T   L   F   L   Y   F   L   A   T   N   H   Y   255
                                         TM3
TGG ATC CTG GTG GAA GGC CTC TAC CTG CAC AGC CTG ATC TTC ATG GCC TTC  816
 W   I   L   V   E   G   L   Y   L   H   S   L   I   F   M   A   F   272

CTC TCC AAC AAG AAC TAC CTG TGG GTC CTC ATC ATC ATT GGC TGG GGT CTC  867
 L   S   N   K   N   Y   L   W   V   L   I   I   I   G   W   G   L   289
                                                TM4
CCT GCT GTG TTT GTG TCC ATC TGG GCC AGC GTC AGG GCC TCC CTG GCA GAC  918
 P   A   V   F   V   S   I   W   A   S   V   R   A   S   L   A   D   306

ACA CAG TGC TGG GAC CTC AGC GCA GGG AAC ATG AAG TGG ATT TAC CAG GTC  969
```

```
 T   Q   C   W   D   L   S   A   G   N   M   K   W   I   Y   Q   V   323
                                                     TM5
CCC ATC TTG GCT GCC GTT GTG GTG AAC TTC TTC CTC TTC CTC AAC ATC GTG  1020
 P   I   L   A   A   V   V   V   N   F   F   L   F   L   N   I   V   340

CGG GTG CTG GCC TCC AAG CTC TGG GAG ACG AAC ACG GGG AAG CCA GAC CCA  1071
 R   V   L   A   S   K   L   W   E   T   N   T   G   K   P   D   P   357

CGG CAG CAG TAC AGG AAG CTG CTG AAG TCC ACG CTG GTG CTG ATG CCG CTT  1122
 R   Q   Q   Y   R   K   L   L   K   S   T   L   V   L   M   P   L   374
                                             TM6
TTT GGA GTG CAC TAC GTG GTG TTC ATG GCC ATG CCC TAC ACC GAA GTC TCC  1173
 F   G   V   H   Y   V   V   F   M   A   M   P   Y   T   E   V   S   391

GGG GTC CTG TGG CAG ATC CAG ATG CAT TAT GAG ATG CTC TTT AAC TCC TCT  1224
 G   V   L   W   Q   I   Q   M   H   Y   E   M   L   F   N   S   S   408
                                                 TM7
CAG GGT TTC TTT GTG GCT TTT ATC TAC TGC TTT TGC AAT GGG GAG GTG CAG  1257
 Q   G   F   F   V   A   F   I   Y   C   F   C   N   G   E   V   Q   425

GCA GAG ATT AAA AAA GCC CAT TTT CGG AGA AGC CTG GCG TTG GAC TTC AAG  1326
 A   E   I   K   K   A   H   F   R   R   S   L   A   L   D   F   K   441

CAG AAG GCG CGT GCC AGC AGC GCA GCA GGG AGC TGC TGT TAT GGT GGG CTG  1377
 Q   K   A   R   A   S   S   A   A   G   S   C   C   Y   G   G   L   459

ATG TCC CAC GGC ACC ACG AAC TTC AGT GTG AGC CTG ACA GGG CGA GGG CCG  1428
 M   S   H   G   T   T   N   F   S   V   S   L   T   G   R   G   P   476

GGG GGC ACA CAG CCC CGG GGG CTG CTC CTC CCT GCC CGT GGC AGC CTG CCA  1479
 G   G   T   Q   P   R   G   L   L   L   P   A   R   G   S   L   P   493

GGC TAC ACC CCC AGC TCC TGT GCT GCA GAC CTT TTG CCC CAC CTG ACG CAG  1530
 G   Y   T   P   S   S   C   A   A   D   L   L   P   H   L   T   Q   510

GAG ATG AGT CAG AAA ACC TGC GGG GAA AAC ACT GTG GGC TCA AAA GAC CCC  1581
 E   M   S   Q   K   T   C   G   E   N   T   V   G   S   K   D   P   527
                                                  PTH3Rfinalrv
GAT GAG AGT CAC CCC AAC CCG AAC AAA GAG CTG GAG ACG ATG CTA TGA      1626
 D   E   S   H   P   N   P   N   K   E   L   E   T   M   L   *       542
```
