# Supplementary figures and images for: Functional characterization and evolution of PTH/PTHrP receptors: insights from the chicken
Source: BMC Evol Biol. 2012 Jul 6;12:110. doi: 10.1186/1471-2148-12-110 (PMC3483286; doi:10.1186/1471-2148-12-110)

Lamprey

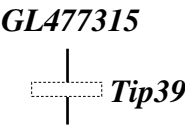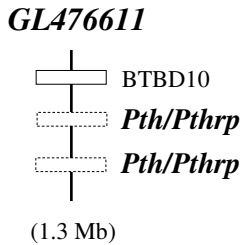

Zebrafish

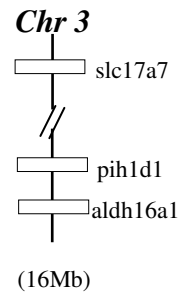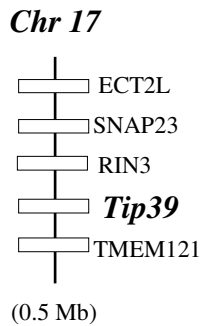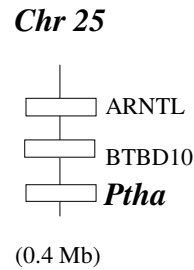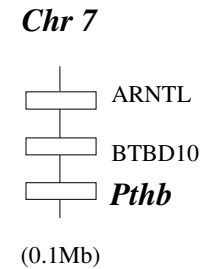

Xenopus

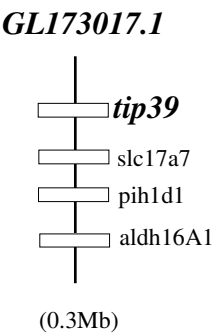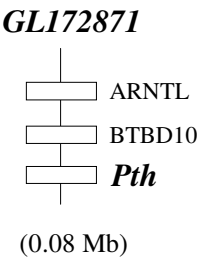

Human

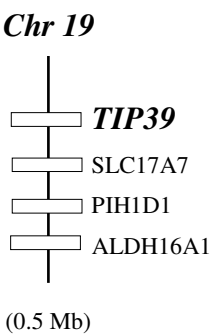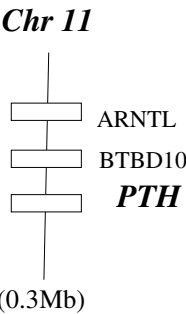

Supplement: Additional file 3 — Comparison of the vertebrate TIP39 and PTH gene environments. Only homologue genes are represented with the exception of the zebrafish TIP39 gene environment to demonstrate the lack of gene synteny with other vertebrate homologue regions. Dashed boxes represent putative gene locus in lamprey. [file 1471-2148-12-110-S3.pdf]

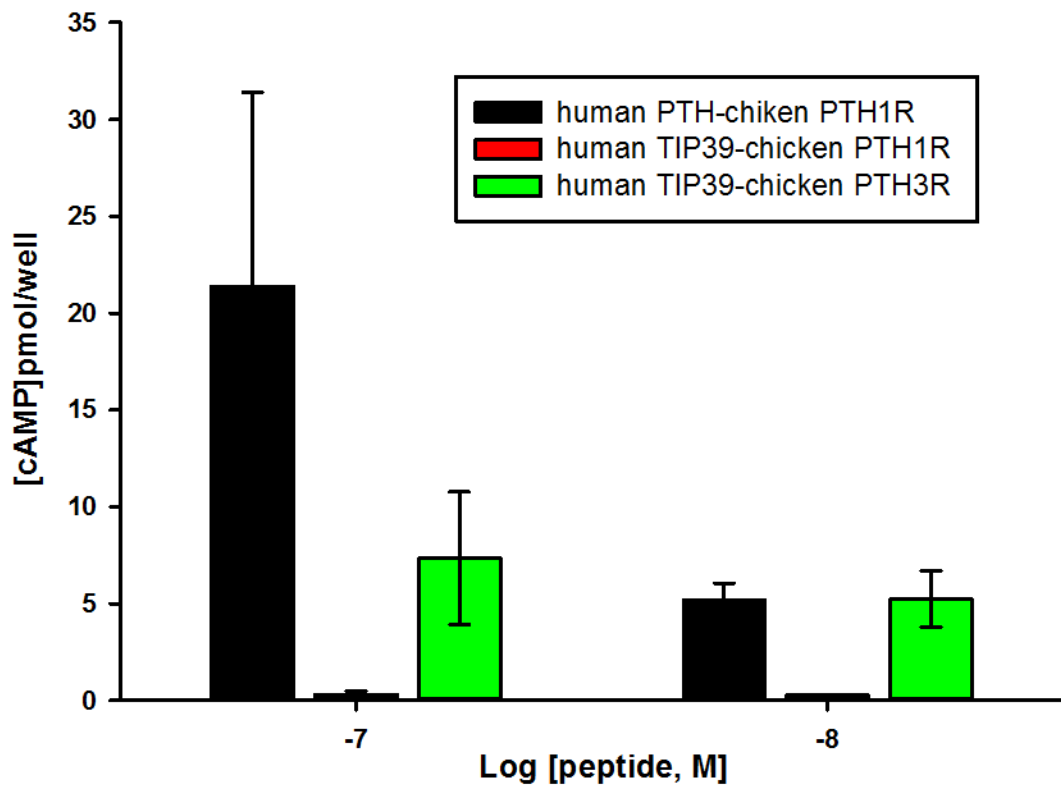

Supplement: Additional file 4 — Accumulation of cAMP in HEK293 cells transfected with chicken PTH1R and PTH3R. Human PTH and TIP39 peptides were used at 10 nM and 100 nM. Values represent means ± SEM of a single experiment carried out in triplicate. [file 1471-2148-12-110-S4.pdf]
